# Intentions to use maternity waiting homes and associated factors in Northwest Ethiopia

**DOI:** 10.1186/s12884-020-02982-0

**Published:** 2020-05-11

**Authors:** Mekonen Endayehu, Mezgebu Yitayal, Ayal Debie

**Affiliations:** 1 East Bellesa District Health Office, North Gondar Zone, Amhara National Regional Health Bureau, Bahir Dar, Ethiopia; 2 Department of Health Systems and Policy, Institute of Public Health, College of Medicine and Health Sciences, University of Gondar, P. O. Box, 196 Gondar, Ethiopia

**Keywords:** Maternity waiting home, Northwest Ethiopia

## Abstract

**Background:**

Maternity Waiting Homes (MWHs) are residential facilities located within hospitals or health centers to accommodate women in their final weeks of pregnancy to bridge the geographical gap in obstetric care. Little is known, however, about women’s intentions to use MWHs. Thus, this study aimed to assess pregnant women’s intentions to use MWHs and associated factors in East Bellesa district, northwest Ethiopia.

**Methods:**

A community-based cross-sectional study was conducted among 525 pregnant women in East Bellesa district from March to May 2018. Study participants were selected using systematic random sampling. Binary logistic regression was used for analysis. Adjusted Odds Ratio (aOR) with 95% Confidence Interval (CI), and *p*-value < 0.05 were used to identify factors associated with intentions to use MWHs.

**Results:**

In the study area, 326/499 (65.3%) pregnant women had the intention to use MWHs. Pregnant women who had good knowledge about maternal healthcare and obstetric complications (aOR 6.40; 95% CI 3.6–11.5), positive subjective norms related to women’s perceptions of social pressure (aOR 5.14; 95% CI 2.9–9.2), positive perceived behavioral control of women on the extent to which women feel confident (aOR 4.74; 95% CI 2.7–8.4), rich wealth status (aOR 4.21; 95% CI 2.1–8.4), women who decided by themselves to use maternal services (aOR 2.74; 95% CI 1.2–6.2), attended antenatal care (aOR 2.24; 95% CI 1.2–4.1) and favorable attitudes towards women’s overall evaluation of MWHs (aOR 1.86; 95% CI 1.0–3.4) had higher odds of intentions to use MWHs.

**Conclusion:**

Two thirds (65.3%) of pregnant women had intentions to use MWHs. Factors such as women’s knowledge, subjective norms related to women’s perceptions of social pressure, perceived behavioral control of women on the extent to which women feel confident to utilize, and wealth status, decision-making power, attending antenatal care and attitude towards women’s overall evaluation of MWHs were significantly associated with the intention to use MWHs. Therefore, improving women’s awareness by providing continuous health education during antenatal care visits, devising strategies to improve women’s wealth status, and strengthening decision-making power may enhance their intention to use MWHs.

## Background

Maternity Waiting Homes (MWHs) are residential facilities located within hospitals or health centers to accommodate women in their final weeks of pregnancy and a strategy to “bridge the geographical gap” in obstetric care between rural areas with poor access to functioning facilities, and urban areas where maternity services are available. MWHs offer a low-cost way to bring women closer to obstetric care as one component of a comprehensive package of essential obstetric services [1]. MWHs have been endorsed by the World Health Organization (WHO) as one component of a comprehensive package to reduce maternal morbidity and mortality [2]. Maternal deaths around the world dropped from about 532,000 in 1990 to an estimated 303,000 at the end of the Millennium Development Goals (MDGs) [3]. Between 1990 and 2015 the Maternal Mortality Ratio (MMR) has been reduced from 385 to 216 per 100,000 live births globally and from 987 to 546 in sub-Saharan Africa (SSA) [4]. The 2016 Ethiopian Demographic Health Survey (EDHS) reported a MMR of 412 per 100,000 live births [5]. In low-and middle-income countries, particularly SSA countries, MMRs are nearly 20 times higher than those in high-income countries [4].

Pregnancy-related maternal mortality is usually affected by the “Three phases of Delay”: first phase delay to decide to seek care in the community, second phase delay to reach appropriate facilities, and third phase delay in the provision of adequate care or receiving adequate care after reaching facilities [6]. Though MWHs have existed in Ethiopia for more than three decades, it has been limited mainly to some hospitals making these inaccessible for most pregnant women [7, 8]. The recent expansion of MWHs to health centers is a breakthrough to bridge the geographic barriers to skilled care. MWHs are built to reduce second phase delays in reaching health facilities in time and it is an institution within easy reach of emergency obstetric and newborn care facilities, where women with high-risk pregnancies await the onset of labor during the final weeks of pregnancy [8, 9].

Cost, distance, and time needed to access care are major barriers to the effective utilization of maternal and child health care in poor and marginalized areas [10]. Available data from a rapid assessment in Eritrea from September 2006 to August 2007 show that a case fatality rate of 1.9% among women who gave birth before the introduction of MWH, but no maternal mortality was recorded after its introduction from May 2008 to April 2009 [11]. Institutional birth rates also increased with 56% in Eritrea after introducing MWHs [11].

A comparative study in Attat and Butajira hospitals showed that pregnancy outcomes among those using MWHs were better than outcomes among non-users [12]. The Ethiopian Ministry of Health (MOH) has made MWHs available in all health facilities throughout the country since 2015. Even though MWHs help to improve maternal health care, costs, distance, and time-related barriers are some of the challenges in using MWHs [4, 13]. In many health facilities, costs for MWHs have been covered with the support of the community, contributing food, and money for women staying in MWHs [13]. Poor accessibility, financial unaffordability, lack of transport and women’s inability to decide on their own to use MWHs are factors affecting institutional births rates in low-resource countries [14].

Few studies, however, have been conducted in Ethiopia to assess women’s intentions towards MWH’s utilization during their last weeks of pregnancy, and findings of these few showed that only 55.1–57.3% of women have intentions to use MWHs [15, 16]. Therefore, this study aimed to assess intentions to use MWHs and associated factors among pregnant women in East Belessa district, northwest Ethiopia.

## Methods

### Study design and settings

A community-based cross-sectional study was conducted among pregnant women in East Bellesa district, northwest Ethiopia from March to May 2018. The district is in the North Gondar administrative zone which is 720 km far from Addis Ababa (the capital city of Ethiopia). Total population of the district was 154,937 with 140,209 rural and 14,728 urban inhabitants [17]. In the district, 35,889 women of reproductive age are estimated to live. The district has one public primary hospital, five health centers, twenty-three health posts, three private primary clinics, and one drug store. Point of service where women got educated about MWHs were home visits by Health Extention Workers (HEWs), ANC visits (84%), home visits by Health Development Armies (HDAs), women’s conferences and other community events [13]. All health centers and the hospital in the district have MWHs and provide free maternal health care including bed and food services. Many MWHs were built with community support, that usually contributed food items and money for women staying in these MWHs, a workforce to build MWHs, and wood and grass for construction. As a result, most MWHs had no budget allocated from government funds [13].

### Population and sampling procedures

All pregnant women in East Belesa district were the source population, while those who resided in the selected kebeles (the lowest administrative units in the country) were the study population. Participants who were critically ill during data collection were excluded. Men were not the study participants, and not interviewed.

The sample size was calculated using a single population proportion formula with an assumption of a 5% margin of error, 95% CI, 1.5 design effect, 5% non-response rate, and 57.3% proportion (P) of pregnant women intended to use MWHs in Jimma district, Southwest Ethiopia [15]. The sample was 592; however, the estimated number of pregnant women in the study area was below 10,000 i.e. only 4639. As a result, we used finite population correction formula and our final sample size was 525. Two urban and six rural kebeles among 22 kebeles in the district were selected using the lottery method. The sample size was allocated proportionally to the expected number of pregnant women in different kebeles.

Finally, study participants were selected using a systematic sampling technique. Sampling interval was determined by dividing the total expected number of pregnant women by the sample size and the interval was three. The first study participant was selected through the lottery method, and then every third household was included. Furthermore, the next household was considered when there were no pregnant women in the selected household.

Pregnancy was ascertained by maternal self-report and presumptive signs and symptoms of pregnancy such as amenorrhea and/or increased uterine-size. Moreover, the Expected Date of Delivery (EDD) and Gestational Age (GA) were calculated by using the first day of the Last Normal Menstrual Period (LNMP) for women who remembered that and measuring the fundal height to estimate GA for women who did not remember their dates. Based on GA, decisions were made whether the pregnancy was term (> 37 weeks) or not.

### Measurements and variables

Intention to use MWHs was measured using three questions: 1) I plan to use MWH for the last remaining 2–4 weeks of my current pregnancy; 2) I will make my effort to use MWH for my current pregnancy; 3) I intend to use MWH for my current pregnancy. Each question contains five points Likert scales (1 = strongly disagree, 2 = disagree, 3 = neutral, 4 = agree, 5 = strongly agree). As a result, the total score was 3–15, and women who scored ≥60% were considered as women who intended to use MWHs.

Knowledge of women about maternal healthcare and obstetric complications in our study was measured by using eight questions: 1) Have you ever heard about maternity waiting homes in health facilities? 2) Do you know about the danger signs of pregnancy? 3) Do you know your expected date of delivery? 4) Do you know your gestational age for your current pregnancy? 5) Do you know giving birth at home has a risk for women and newborns? 6) Do you know that using an MWH is important for pregnant women who need immediate obstetric care? 7) Do you know about birth preparedness and complication readiness? 8) Are there MWHs in your area? Each question contains 0 = no and 1 = yes. The total score ranged from 0 to 8 and a score of ≥60% was considered as knowledgeable.

Women’s attitudes towards MWHs were measured using four questions: 1) For me using MWH is good; 2) For me using MWH is valuable; 3) For me using MWH is pleasant; 4) For me using MWH is beneficial. Each question contains five points Likert scales (1 = strongly disagree, 2 = disagree, 3 = neutral, 4 = agree, 5 = strongly agree). The total score was 4–20 and women who had scored ≥60% were considered as having favorable attitudes.

Furthermore, subjective norms (SN) towards MWH utilization, related to women’s perceptions of social pressure to utilize MWHs, were measured by using five questions: 1) Most people who are important for me, think that I should use MWH; 2) Whether I use MWH or not is up to me; 3) Most women in my village/neighborhood use MWHs; 4) It is expected from me to use MWH; 5) Most people whose opinions I value, would approve of my using MWH. Each question contains five points Likert scales (1 = strongly disagree, 2 = disagree, 3 = neutral, 4 = agree, 5 = strongly agree). The total score was 5–25 and women who had scored ≥60% were considered as having positive SNs.

Perceived behavioral control (PBC) towards MWHs is an indicator of the extent to which women feel confident to utilize MWHs. PBC was measured by using three questions: 1) For me to use MWH is very easy in our community; 2) If I want, I am confident that I can use MWH in the last 2–4 weeks of my pregnancy; 3) Using MWHs is possible in our set up. Each question contains five points Likert scales (1 = strongly disagree, 2 = disagree, 3 = neutral, 4 = agree, 5 = strongly agree). The total score ranged from 3 to 15 and women who had scored ≥60% were considered as having positive PBC.

Mode of transport to health facilities was assessed through asking mode of transport during obstetric emergencies and women’s responses were categorized as on foot/carried by others, private bus or ambulance. Distance to reach health facilities was measured by asking how long it would take to reach the nearest health center or hospital from their house on foot in minutes and categorized as less than 60 min and 60 min or more.

Obstetric history was assessed by asking previous obstetric characteristics and utilization of antenatal care, place of birth, postnatal care, stillbirth history, and facility type. A residence was considered to be urban if women for the last 6 months stayed in areas identified by the municipality as a town.

Wealth status was measured by assessing any property of households using principal component analysis. Wealth status was categorized as low (poor), medium, and high (rich). The source of information about MWHs has been assessed by using their most frequent source of information.

Intention to use MWHs was the dependent variable whereas factors such as socio-demographic and economic factors, maternal knowledge, attitude, subjective norm, perceived behavioral control, transportation, distance, and obstetric history were independent variables of the study.

### Data collection tools and procedures

Data were collected using interviewer-administered structured questionnaires adapted from previous studies [15, 16, 18]. The questionnaire was first prepared in English and translated to Amharic, and finally back to English to ensure consistency. Six diploma graduated midwives were recruited as data collectors and two Bachelor of Science (BSc) graduated midwives as supervisors. Two days of training was given for data collectors and supervisors on basic techniques of data collection. A pre-test was done among 26 women in west Bellesa district who had similar setups as the study area and some modifications such as language editing for ambiguous questions were made. Supervisors followed up the process of data collection daily, checked data for completeness and consistency, and communicated with the principal investigator to be able to take immediate measures.

### Data management and analysis

Data were analyzed using EPI -INFO version 7.1software and exported to SPSS version 20. To reduce missing data, we used double data entry methods. If we faced missing data, data were re-entered through retrieving data from hard copies. Data cleaning and close supervision were also conducted daily. Questionnaires, however, with incomplete and unretrievable data were considered as non-response. Completeness of the data was checked by running frequencies and cross-tabulation. Recoding, valuing, transforming, computing, and categorizing variables were done before analysis. Binary logistic regression was performed and those variables having *p*-values < 0.2 were fitted into multivariable logistic regression analysis. In multivariable logistic regression analysis, adjusted Odds Ratio (aOR) with 95% CI and p-value < 0.05 were used to identify factors associated with intentions to use MWHs.

## Results

### Socio-demographic characteristics

A total of 499 (95%) pregnant women participated in the study. Almost half (233/499; 47.9%) of the participants were in the age range of 25–34 years, the majority was unable to read and write (410/499; 82.2%), housewives (460/499; 92.2%), married (455/499; 91.2%), Orthodox Christians (481/499; 96.4%), rural residents (383/499; 76.8%) and (166/499; 33.3%) had the poorest wealth status (Table 1). The study also revealed that 363/499 (72.7%) husbands were unable to read and write, and 442/499 (88.6%) were farmers. Twenty-one percent (105/499) of pregnant women made maternal health care decisions by themselves, 214/499 (42.9%) of them had good knowledge about maternal health care and pregnancy-related complications, 167/499 (33.5%) took more than an hour to reach the nearest health facility, and coming on foot or carried by others was means of transport for 232/499 (46.5%) of participants during emergencies. Health workers were the source of information about maternal health care for 347/499 (69.5%) women (Table 1).

**Table 1:** Socio-demographic characteristics of pregnant women in East Bellesa district northwest Ethiopia, 2018

Variables	Category	Frequency	Percent (%)
Age of women in years	< 25	245	49.1
	25–34	233	46.7
	≥ 35	21	4.2
Residence	Rural	383	76.8
	Urban	116	23.2
Maternal education	Unable to read and write	410	82.2
	Primary school	65	13.0
	Secondary and above	24	4.8
Maternal occupation	Housewives	460	92.2
	Merchants	26	5.2
	Government employees	13	2.6
Current marrital status	Unmarried	44	8.8
	Married	455	91.2
Religion	Orthodox	481	96.4
	Muslim	18	3.6
Husband’s education	Unable to read and write	363	72.7
	Primary school	93	18.6
	Secondary and above	43	8.6
Husband’s occupation	Farmer	442	88.6
	Merchants	37	7.4
	Government employee	20	4.0
Family size	< 3	183	36.7
	3–4	172	34.5
	≥ 5	144	28.9
Wealth status	Poor	166	33.3
	Medium	175	35.1
	Rich	158	31.7
Decision maker	Partner	139	27.9
	Together	255	51.1
	Woman	105	21.0
Time to reach nearest health facility	Less than 60 min	332	66.5
	60 or more minutes	167	33.5
Transport during emergency	On foot/carried by others	232	46.5
	Private bus	56	11.2
	Ambulance	211	42.5
Source of information	Health care providers	347	69.5
	Health development army	57	11.4
	Mass media	95	19.0
Knowledge of mothers	Not knowledgeable	285	57.1
	Knowledgeable	214	42.9

### Obstetric characteristics

Among participants, 436/499 (87.4%) women had at least one previous birth (multigravida), 76/499 (15.2%) had five or more pregnancies, 363/499 (72.7%) had started ANC, and 219/436 (50.2%) had PNC for their previous births. Besides, 24/436 (5.5%) women had a previous history of MWH utilization and 19 of these women were referred by Health Extension Workers to MWHs, out of whom seven stayed more than 2 weeks in a MWH (Table 2).

**Table 2:** Obstetric histories in East Bellesa district northwest Ethiopia, 2018

Variables	Category	Frequency	Percent (%)
Gravidity	< 5	423	84.8
	**≥** 5	76	15.2
ANC visit	No	136	27.3
	Yes	363	72.7
Number of ANC visits (*n* = 363)	1	71	19.6
	2	129	35.5
	3	119	32.8
	**≥** 4	44	12.1
Type of HF for ANC (*n* = 363)	Health post	33	9.1
	Health center	273	75.2
	Hospital	57	15.7
Gave childbirth previously	No	63	12.6
	Yes	436	87.4
Place of last birth (*n* = 436)	Health facility	156	35.8
	Home	280	64.2
Stillbirth	No	457	91.6
	Yes	42	8.4
Abortion	No	452	90.6
	Yes	47	9.4
Number of children	0	65	13.0
	1–4	413	82.8
	**≥**5	21	4.2
Postnatal care (*n* = 436)	No	217	49.8
	Yes	219	50.2
Use MWH previously (*n* = 436)	No	412	94.5
	Yes	24	5.5
Referred by (*n* = 24)	Self	5	20.8
	HEWs	19	79.2
Length of stay at MWH (in weeks) (*n* = 24)	≤ 2	17	70.8
	> 2	7	29.2
Family attendant at MWH (*n* = 24)	No one	3	12.5
	Husband and his families	11	45.8
	Women’ families	10	41.7
Financial supporter in MWH (*n* = 24)	Self	6	25.0
	Husband / partner	2	8.3
	Health facility	16	66.7

### Intentions to use MWHs and behavioral characteristics

**Fig. 1:**
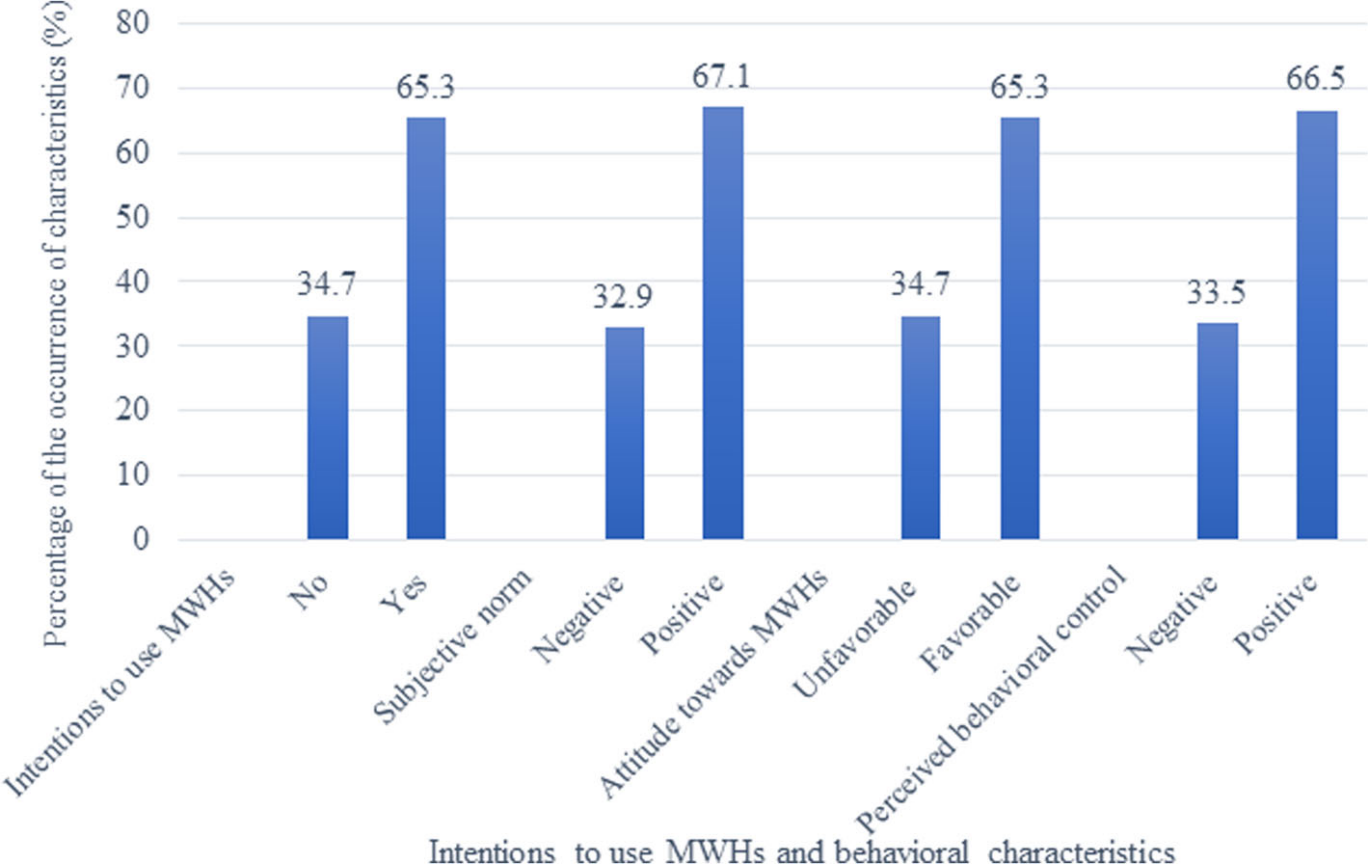
Intentions to use and behavioural characterstics towards MWHs among pregnant women in East Belessa district northwest Ethiopia, 2018

The study revealed that 326/499 (65.3%) women intended to use MWHs. Moreover, pregnant women had positive subjective norms (335/499; 67.1%), perceived behavioral control (332/499; 66.5%), and favorable attitudes (326/499; 65.3%) towards MWHs (Fig. 1).

### Factors associated with intentions to use MWHs

Women had higher odds of intending to use MWHs if they had attended ANC compared to those who did not (aOR 2.24; 95% CI 1.2–4.1). Women who made health care decisions by themselves (aOR 2.74; 95% CI 1.2–6.2) and were categorized as having medium (aOR 2.38; 95% CI 1.3, 4.5) and rich wealth status (aOR 4.21; 95% CI 2.1–8.4) were more likely to use MWHs compared to poor women (Table 3).

**Table 3:** Factors associated with women’s intentions to use MWHs in East Bellesa district northwest Ethiopia, 2018

Variables	Category	Intention to use MWH		cOR (95%CI)	aOR (95% CI)
		Yes	No		
Age of women in years	Less than 30	283	147	1	1
	30 or more	43	26	0.86 (0.5,1.5)	0.97 (0.6,2.9)
Current marital status	Unmarried	29	15	1	1
	Married	297	158	0.97 (0.5,1.9)	0.60 (0.2,1.6)
Maternal education	Unable to read & write	260	150	1	1
	Primary school	48	17	1.63 (0.9,2.9)	1.78 (0.8,4.0)
	Secondary & above	48	6	1.73 (0.7,4.5)	1.56 (0.4,6.1)
Gravidity	< 5	269	154	1	1
	≥5	57	19	1.72 (1.0,3.0)	1.61 (0.6,4.2)
Family size	< 3	120	63	1	1
	3–4	114	58	1.03 (0.7,1.6)	1.02 (0.5,2.0)
	≥ 5	92	52	0.93 (0.6,1.5)	0.85 (0.5,1.8)
ANC visit	No	77	59	1	1
	Yes	249	114	1.67 (1.1,2.5)	2.24 (1.2,4.1) *
Decision-maker	Husband	74	65	1	1
	Together (both)	171	84	1.79 (0.8,3.4)	1.52 (0.4,2.6)
	Herself	81	24	2.96 (1.9,6.5)	2.74 (1.2,6.2) *
Transport during emergency	Foot / carried by others	135	95	1	1
	Private transport	36	20	1.25 (0.7,2.3)	1.22 (0.5,3.0)
	Ambulance	153	58	1.83 (1.2,2.7)	1.37 (0.8,2.4)
Time to nearest health facility	Less than 60 min	215	117	1	1
	60 or more minutes	111	56	1.08 (0.7,1.6)	1.04 (0.5,1.4)
Subjective norm	Negative	52	112	1	1
	Positive	274	61	9.68 (6.3,14.9)	5.14 (2.9,9.2) *
Attitudes towards MWH	Unfavorable	56	68	1	1
	Favorable	270	105	3.12 (2.1,4.8)	1.86 (1.0,3.4) *
Perceived behavioural control	Negative	59	108	1	1
	Positive	267	65	7.52 (5.0,11.4)	4.74 (2.7,8.4) *
Wealth status	Poor	91	75	1	1
	Medium	116	59	1.62 (1.1,2.5)	2.38 (1.3,4.5) *
	Rich	119	39	2.51 (1.6,4.0)	4.21 (2.1,8.4) *
Knowledge of women	Not knowledgeable	140	145	1	1
	Knowledgeable	186	28	6.88 (4.3,10.9)	6.40 (3.6,11.5) *

*significant at p-value < 0.05

Women who had good knowledge about maternal health care and obstetric complications (aOR 6.40; 95% CI 3.6–11.5), positive SNs (aOR 5.14; 95% CI 2.9–9.2), positive PBC (aOR 4.74; 95% CI 2.7–8.4), and favorable attitudes (aOR 1.86; 95% CI 1.0–3.4) were more likely to use MWHs compared to those without those risk factors (Table 3).

## Discussion

Our study revealed that 65.3% of 499 women had intentions to use MWHs. This percentage is lower than in Wolaita Sodo town, Ethiopia (75.5%), and Nyanza Province, Kenya (76.9%) [19]. It is higher, however, than in Jimma district, Ethiopia (57.3%) [15], four districts of Eastern Gurage Zone, Southern Ethiopia (55.1%) [16], and rural Kenya (45%) [20]. Possible explanations may be differences in study participants, areas, period, and design. In some areas, the study was only conducted in health facilities among ANC attendants or urban dwellers who may have a better understanding of maternal health issues. On the other hand, some studies were done at the community level, particularly in rural settings. These women may have lower levels of awareness due to a lack of information about maternal health care, including MWHs.

Women who attended ANC had higher odds of intentions to use MWHs than participants who did not. This finding is supported by studies in Tsegedie, North Gondar, Bahir Dar town, and Tigray region, Ethiopia, [21–24]. The possible explanation may be fear of complications during childbirth as a result of education by midwives to use MWHs during ANC visits. Even women who attended ANC in health facilities without MWHs were advised to go and wait for birth in district hospitals or health centers with MWHs. Women attending ANC have the chance to familiarize themselves with the health facilities’ environments. This may reduce unnecessary fear and stress related to institutional birth. Moreover, women may also be better informed about danger signs and obstetric complications that may occur during pregnancy and childbirth [25].

Odds of intentions to use MWHs among respondents who decided by themselves were higher than in the case decisions made by their husbands. This is supported by studies in rural health centers in Amhara, Oromia, Southern Nations Nationalities and People (SNNP), and Tigray regions Ethiopia and Kenya [13, 20]. A possible explanation might be that husbands want their wives to stay at home until the expected date of delivery because of heavy workload and family care. Refusal of admission by husbands may be because of a lack of other adult caretakers at home. This could be the reason why women in remote areas are not motivated to come to MWHs because the whole household is dependent on women for all household support [13]. On the contrary, studies in Attat hospital, Ethiopia [26], rural southern Egypt (Upper Egypt) [27], rural Zambia [28] and Sierra Leone [29] reported that husbands played important roles to encourage their wives to use MWHs. They possibly have positive attitudes towards MWHs and perceived benefits from using MWHs, including mitigating long distances and improving access to facility-based delivery care.

Women with a medium and rich wealth status had higher odds of intentions to use MWHs compared to women with poor wealth. This is in line with studies in rural Timor Leste, Mayan women in Guatemala, remote areas of Nepal, and Southern Lao PDR [30–34]. Possible explanations might be the costs of public transport and other costs associated with the use of MWHs. Availability of free ambulance services, lessening of costs associated with using MWHs, and subsequent institutional birth are important strategies for enhancing MWH utilization.

Women with knowledge about maternal services and obstetric complications, with favorable attitudes towards the overall evaluation of MWHs, positive subjective norms related to women’s perceptions on social pressure and perceived behavioral control on the extent to which they feel confident to utilize MWHs had higher odds of using MWHs compared with their counterparts. The effect of woman’s knowledge on MWHs is in line with studies in Timor Leste, Bangladesh, and Northern Sierra Leone [29, 35–37]. Possible justification might be that women who are well aware of maternal healthcare services and obstetric complications may fear bad outcomes of birth at home. Similarly, women’s favorable attitudes towards MWHs were in agreement with studies in Jimma district, Ethiopia, rural Zimbabwe, and Zambia [15, 28, 38, 39]. This might be due to the wrong perceptions of community members that women admitted to MWHs are lazy or careless to abandon their families [13]. Respondents’ subjective norms were also consistent with findings in Wolaita Sodo town and Jimma district, Ethiopia [15, 18]. Women with positive perceptions of social pressure may possibly have more inclination to utilize maternal healthcare services. Moreover, women’s perceived behavioral control in our study was in line with studies in Jimma district, Ethiopia, and Kalomo, Zambia [15, 40]. Women who have positive perceived behavioral control may feel more confident to utilize MWHs.

Maternal knowledge, subjective norms, and wealth status had the highest odds for intentions to use MWHs. The more favorable attitudes and subjective norms, the greater perceived control resulted in stronger women’s intentions to use MWHs with fewer worries about food shortages and the costs of transport [15, 38].

### Limitation of the study

One limitation of our study was that it could not show any cause-effect relationship. Beliefs of the women towards intentions to use MWHs may not be fully addressed since the study lacked a qualitative approach. We also did not address the needs and beliefs of the women’s partners, indicating gender inequality bias.

## Conclusion

Two third of the women in this study had the intention to use MWHs. Factors such as women’s attitudes, subjective norms, perceived behavioral control, decision-making power, knowledge, wealth status, and ANC utilization were significantly associated with intentions to use MWHs. Improving women’s attitudes and behavioral perceptions through awareness creation by continuous health education during ANC visits, devising strategies to improve wealth status, and strengthening women’s decision-making power in the household may enhance women’s intentions to use MWHs.

## Data Availability

Datasets supporting the conclusions of this article are available upon request to the corresponding author. Due to data protection restrictions and participant confidentiality, we do not make participants’ data publicly available.

## Abbreviations

ANC: Ante-natal care
BP: Birth place
EDHS: Ethiopian demographic health survey
HC: Health center
HF: Health facility
HP: Health post
MNH: Maternal and newborn health
MMR: Maternal mortality ratio
MWH: Maternity waiting home
PBC: Perceived behavioural control
PNC: Post natal care
RHB: Regional health bureau
SN: Subjective norm
SDA: Skilled delivery attendance
WB: World bank
WHO: World health organization

## References

[1] World Health Organization. WHO recommendations on health promotion interventions for maternal and newborn health. Geneva: WHO; 2015. 26180864

[2] World Health Organization (1996). Maternity waiting homes: a review of experiences.

[3] World Health Organization. Maternal deaths fell 44% since 1990–UN: Report from WHO, UNICEF: UNFPA, World Bank Group, and the United Nations Population Division. Geneva: WHO; 2015.

[4] World Health Organization and UNICEF. Trends in maternal Mortality: 1990-2015: Estimates from WHO, UNICEF, UNFPA, World Bank Group and the United Nations Population Division. Geneva; WHO; 2015.

[5] Central Stastical Agency (CSA). Ethiopia Demographic and Health Survey. Addis Ababa: CSA; 2016.

[6] Thaddeus S, Maine D (1994). Too far to walk: maternal mortality in context. Soc Sci Med.

[7] Kelly J, Kohls E, Poovan P, Schiffer R, Redito A, Winter H, MacArthur C (2010). The role of a maternity waiting area (MWA) in reducing maternal mortality and stillbirths in high-risk women in rural Ethiopia. BJOG..

[8] Gaym A, Pearson L, Soe K (2012). Maternity waiting homes in Ethiopia--three decades experience. Ethiop Med J.

[9] World Health Organization: Making pregnancy safer: the critical role of the skilled attendant. *a joint statement by WHO, ICM and FIGO,*2004.

[10] World Health Organization: Health in 2015: from MDGs, millennium development goals to SDGs, sustainable development goals. 2015.

[11] Andemichael G, Haile B, Kosia A, Mufunda J (2009). Maternity waiting homes: a panacea for maternal/neonatal conundrums in Eritrea. JEMA.

[12] Braat F, Vermeiden T, Getnet G, Schiffer R, van den Akker T, Stekelenburg J (2018). Comparison of pregnancy outcomes between maternity waiting home users and non-users at hospitals with and without a maternity waiting home: retrospective cohort study. Int Health.

[13] Tiruneh GT, Taye BW, Karim AM, Betemariam WA, Zemichael NF, Wereta TG, Lemango ET (2016). Maternity waiting homes in rural health centers of Ethiopia: the situation, women’s experiences and challenges. Ethiop J Health Dev.

[14] van Lonkhuijzen L, Stekelenburg J, van Roosmalen J (2012). Maternity waiting facilities for improving maternal and neonatal outcome in low-resource countries. Cochrane Database Syst Rev.

[15] Endalew GB, Gebretsadik LA, Gizaw AT (2016). Intention to use maternity waiting home among pregnant women in Jimma District, Southwest Ethiopia. GJMR-K.

[16] Vermeiden T, Braat F, Medhin G, Gaym A, van den Akker T, Stekelenburg J (2018). Factors associated with intended use of a maternity waiting home in southern Ethiopia: a community-based cross-sectional study. BMC Pregnancy Childbirth.

[17] Amhara National Regional State Finace Bureau: population estimation of East Bellesa district,. 2017.

[18] Lera T, Admasu B, Dirar A (2017). Intention to use institutional delivery and associated factors among ANC attendants in Wollaita Soddo town, southern Ethiopia: a cross-sectional community based study, application of theory of planned behavioral model. Am J Public Health Res.

[19] Creanga AA, Odhiambo GA, Odera B (2016). Pregnant Women’s intentions and subsequent behaviors regarding maternal and neonatal service utilization: results from a cohort study in Nyanza Province, Kenya. PLoS One.

[20] Mramba L, Nassir FA, Ondieki C, Kimanga D (2010). Reasons for low utilization of a maternity waiting home in rural Kenya. Int J Gynaecol Obstet.

[21] Tsegay Y, Gebrehiwot T, Goicolea I, Edin K, Lemma H, San Sebastian M (2013). Determinants of antenatal and delivery care utilization in Tigray region, Ethiopia: a cross-sectional study. Int J Equity Health.

[22] Hailu D, Berhe H (2014). Determinants of institutional childbirth service utilisation among women of childbearing age in urban and rural areas of Tsegedie district, Ethiopia. Midwifery.

[23] Abeje G, Azage M, Setegn T (2014). Factors associated with institutional delivery service utilization among mothers in Bahir Dar City administration, Amhara region: a community based cross sectional study. Reprod Health.

[24] Worku AG, Yalew AW, Afework MF (2013). Maternal complications and women's behavior in seeking care from skilled providers in North Gondar. Ethiopia PLoS One.

[25] Fekadu GA, Kassa GM, Berhe AK, Muche AA, Katiso NA (2018). The effect of antenatal care on use of institutional delivery service and postnatal care in Ethiopia: a systematic review and meta-analysis. BMC Health Serv Res.

[26] Vermeiden T, Schiffer R, Langhorst J (2018). Facilitators for maternity waiting home utilisation at Attat hospital: a mixed-methods study based on 45 years of experience. Tropical Med Int Health.

[27] Ohashi A, Higuchi M, Labeeb SA, Mohamed AG, Chiang C, Aoyama A (2014). Family support for women’s health-seeking behavior: a qualitative study in rural southern Egypt (upper Egypt). Nagoya J Med Sci.

[28] Sialubanje C, Massar K, van der Pijl MS, Kirch EM, Hamer DH, Ruiter RA (2015). Improving access to skilled facility-based delivery services: Women’s beliefs on facilitators and barriers to the utilisation of maternity waiting homes in rural Zambia. Reprod Health.

[29] Kyokan M, Whitney-Long M, Kuteh M, Raven J (2016). Community-based birth waiting homes in northern Sierra Leone: factors influencing women's use. Midwifery..

[30] Wild K, Barclay L, Kelly P, Martins N (2012). The tyranny of distance: maternity waiting homes and access to birthing facilities in rural Timor-Leste. Bull World Health Organ.

[31] Ruiz MJ, van Dijk MG, Berdichevsky K, Munguía A, Burks C, García SG (2012). Barriers to the use of maternity waiting homes in indigenous regions of Guatemala: a study of users' and community members' perceptions. Cult Health Sex.

[32] Schooley J, Mundt C, Wagner P, Fullerton J, O’Donnell M (2009). Factors influencing health care-seeking behaviours among Mayan women in Guatemala. Midwifery..

[33] Shrestha SD, Rajendra PK, Shrestha N. Feasibility study on establishing maternity waiting homes in remote areas of Nepal. Regional Health Forum. 2007;11:33–8.

[34] Eckermann E, Deodato G (2008). Maternity waiting homes in southern Lao PDR: the unique ‘silk home’. J Obstet Gynaecol Res.

[35] Gabrysch S, Campbell OM (2009). Still too far to walk: literature review of the determinants of delivery service use. BMC Pregnancy Childbirth..

[36] Islam N, Islam MT, Yoshimura Y (2014). Practices and determinants of delivery by skilled birth attendants in Bangladesh. Reprod Health.

[37] Wild K, Barclay L, Kelly P, Martins N (2010). Birth choices in Timor-Leste: a framework for understanding the use of maternal health services in low resource settings. Soc Sci Med.

[38] Glanz K, Rimer BK, Viswanath K. Health behavior and health education: theory, research, and practice. San Francisco: Jossey-Bass; 2008.

[39] Millard L (1991). Antenatal village stay and pregnancy outcome in rural Zimbabwe. Cent Afr J Med.

[40] Sialubanje C, Massar K, Hamer DH, Ruiter RA (2014). Personal and environmental predictors of the intention to use maternal healthcare services in Kalomo, Zambia. Health Educ Res.

